# Non-surgical vs. surgical treatment of distal radius fractures: a meta-analysis of randomized controlled trials

**DOI:** 10.1186/s12893-024-02485-1

**Published:** 2024-07-10

**Authors:** Chaohua Zhu, Xue Wang, Mengchao Liu, Xiaohui Liu, Jia Chen, Guobin Liu, Gang Ji

**Affiliations:** 1 Department of Orthopedics, The First hospital of Hebei Medical University, 89 Donggang Road, Shijiazhuang, 050031 China; 2 Tianjin Key Laboratory of Bone Implant Interface Functionalization and Personality Research, Just Medical Equipment (Tianjin) Co., Ltd, Tianjin, 300190 China

**Keywords:** Distal radius fracture, Nonsurgical, Surgical, Treatment, Randomized controlled trials, Meta-analysis

## Abstract

**Purpose:**

To compare the clinical outcomes between nonsurgical and surgical treatment of distal radius fracture.

**Methods:**

We performed a systematic literature search by using multiple databases, including Medline, PubMed, and Cochrane. All databases were searched from the earliest records through February 2023. The study compared nonsurgical versus surgical treatment of distal radius fractures and included only randomized controlled trials (RCTS).

**Results:**

There were seventeen randomized controlled trials retrieved. A total of 1730 patients were included: 862 in the nonsurgical group and 868 in the surgical group. The results showed a significant reduction in DASH score with surgical treatment (*WMD* 3.98, 95% *CI* (2.00, 5.95), *P* < 0.001). And in grip strength (%), the results showed a significant improvement in surgical treatment compared with non-surgical treatment (*WMD* − 6.60, 95% *CI* (-11.61, -1.60), *P* = 0.01). There was significant difference in radial inclination, radial length, volar title, range of wrist pronation, range of wrist supination. However, no difference in radial deviation, ulnar deviation, ulnar variance, range of wrist extension and range of wrist flexion was observed.

**Conclusions:**

The results of this meta-analysis suggest that some patients with surgical treatment of distal radius fractures not only improved the grip strength (%), decreased the DASH score, but also improved the range of wrist pronation and the range of wrist supination compared with nonsurgical treatment. Based on the present meta-analysis, we suggest that some patients with surgical treatment might be more effective in patients with distal radius fracture.

**Supplementary Information:**

The online version contains supplementary material available at 10.1186/s12893-024-02485-1.

## Introduction

As the most common orthopedic injuries, distal radius fractures (DRFs) can cause wrist joint disability with a peak incidence in persons 18–25 years of age and second peak in persons older than 65 years [23]. The management of distal radius fractures consists of non-surgical or surgical treatment. However, there is no consistent evidence regarding the optimal treatment.

Various methods have been described to improve fracture stability and prevent the loss of reduction of the distal radius [1, 3, 7, 12, 18, 20, 33]. However, no consensus has been reached regarding the optimal treatment method when to perform surgery, what type of surgery is best, and what nonsurgical treatment is best for the treatment of DRFs [5, 33]. Several meta-analyses reported that no difference in functional outcome between non-surgical or surgical treatment [8, 15, 22]. Recently, a retrospective cohort study examined distal radius fractures over a 10-year period concluded that there has been a movement toward open reduction and internal fixation among patients with distal radius fracture [2].

The purpose of this study was to perform a systematic review and meta-analysis of randomized controlled trials comparing the outcomes of surgical and nonsurgical management of DRFs.

## Materials and methods

### Literature search strategy

All databases were searched from the earliest records through February 2023. We strictly followed the methods established in the Cochrane Handbook for Systematic Reviews of Interventions 5.0.2, and the Preferred Reporting Items for Systematic Reviews and Meta-Analyses 2020 checklist [24] and ASMTAR 2. Two independent investigators performed an electronic search of the following databases: PubMed, Medline, and the Cochrane Registry of Clinical Trials. Disagreement between the 2 reviewers was addressed by discussion until agreement was reached.

## Literature search and study selection

The search used the following terms and Boolean operators: (((((((((radius fractures[MeSH Terms]) AND distal[Title/Abstract])) OR colles’ fracture[MeSH Terms]) OR wrist injuries[MeSH Terms])) OR ((((((((radius[Title/Abstract]) OR radial[Title/Abstract])) AND distal[Title/Abstract])) AND fractur*[Title/Abstract])) OR ((((colles[Title/Abstract]) OR smith[Title/Abstract]) OR barton[Title/Abstract]) OR wrist[Title/Abstract]))) AND fractur*[Title/Abstract]))))) AND ((((((((surgical procedure, operative[MeSH Terms]) OR fracture fixation[MeSH Terms]) OR orthopedic procedure[MeSH Terms]) OR orthopedics[MeSH Terms])) OR ((((((((((((surg*[Title/Abstract]) OR operat*[Title/Abstract]) OR orthop*[Title/Abstract]) OR pin*[Title/Abstract]) OR nail*[Title/Abstract]) OR screw*[Title/Abstract]) OR plat*[Title/Abstract]) OR rod*[Title/Abstract]) OR wire*[Title/Abstract]) OR fix*[Title/Abstract]) OR ORIF[Title/Abstract]) OR ExFix[Title/Abstract]))) AND ((((conservative treatment[MeSH Terms]) OR physical therapy modalities[MeSH Terms])) OR ((((((((((((((((conserv*[Title/Abstract]) OR conven*[Title/Abstract]) OR non-operat*[Title/Abstract]) OR “non operative“[Title/Abstract]) OR nonoperat*[Title/Abstract]) OR non-surg*[Title/Abstract]) OR “non surgical“[Title/Abstract]) OR nonsurg*[Title/Abstract]) OR cast*[Title/Abstract]) OR splint*[Title/Abstract]) OR brace*[Title/Abstract]) OR bracing[Title/Abstract]) OR plaster[Title/Abstract]) OR bandage*[Title/Abstract]) OR tape*[Title/Abstract]) OR taping[Title/Abstract]))). We applied no restrictions on language or year of publication. Additional relevant studies were identified by perusing the references of retrieved studies and review articles.

The criteria for inclusion of the studies included: (1) randomized controlled trials; (2) The types of fractures include distal radius fractures, as well as distal radius fractures with special names, such as Colles fractures, Smith fractures, Barton fractures, etc. (3) studies that directly compared the nonsurgical and surgical management, with available clinical outcomes. Exclusion criteria were studies where no comparative data were provided.

## Data extraction and outcome measures

Once the studies met the inclusion criteria, data were extracted by 2 reviewers independently. For each trial, data were collected on the following characteristics: patient demographics, study design, number of participants in each treatment group, participants’ age and gender, type of intervention for each group, Disabilities of the Arm, Shoulder and Hand (DASH) scores for study and control groups, functional assessment by grip strength and volar title, ranges of wrist extension, flexion, pronation, and supination, and radial and ulnar deviation, and radial inclination, length, and ulnar variance. If the original data in the original article did not provide information such as mean or standard difference, but we could have obtained it by converting other data provided in the article, we also included such studies. The standard for data conversion we refer to is the Cochrane Handbook for Systematic Reviews of Interventions.

## Quality assessment

The methodological quality of the included trials was evaluated independently by the reviewers using a specific tool for assessing risk of bias, as recommended by the Cochrane Collaboration to assess methodological quality of clinical trials. This comprises a description and judgment for each entry in a “risk of bias” table, where each entry addresses a specific feature of the study.

## Statistical analysis

Statistical analyses were conducted using the software Review Manager 5.3, R 4.2.2 and Stata 16. The treatment effects were expressed as risk ratios (*RR*), with 95% confidence intervals (*CI*) for dichotomous outcomes and weighted mean difference (*WMD*) with 95% CI for continuous outcomes [14]. Heterogeneity was tested using the Chi-square test with significance set at *P* < 0.1 [14]. The I-square test was also used to quantify the effect of heterogeneity with an *I*^*2*^ of 50% or higher representing substantial heterogeneity [25]. If there was no statistical evidence of heterogeneity, a fixed-effects model was used; otherwise, a random-effects model was adopted [14]. If standard deviation was required to be calculated from raw data, SPSS 25.0 software was used.

## Results

**Fig. 1:**
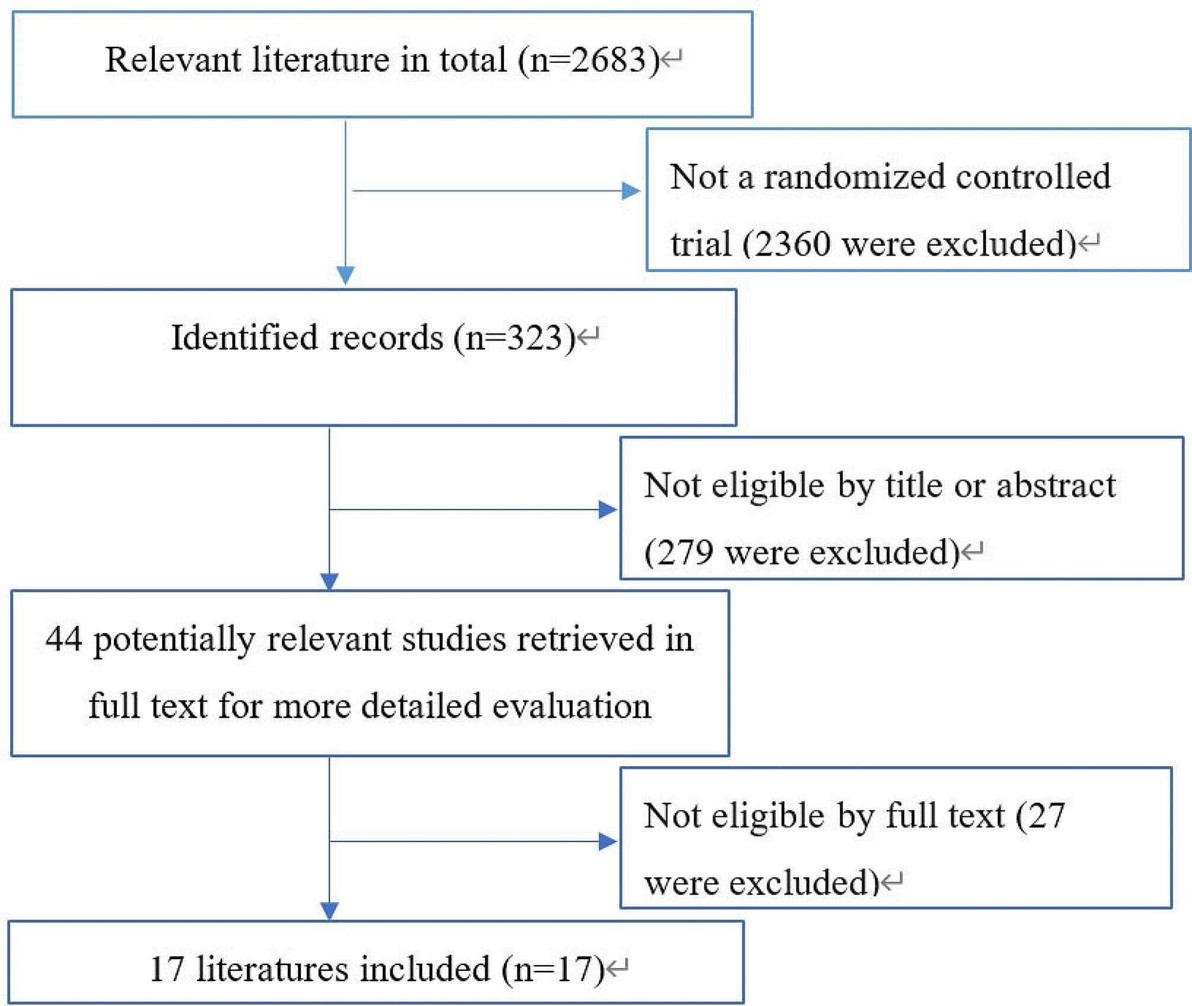
A PRISMA flow chart illustrated the selection of studies included in our systematic review

A flow chart of the study selection process is presented in Fig. 1. An initial search identified 2683 articles from the search protocol, of which 2360 articles were not RCT studies, leaving 323 records available for query. After further evaluation of the titles, text words, and abstracts, 44 potentially relevant studies were selected for full-text examination. Finally, 17 randomized controlled trials were determined as appropriate to match the inclusion criteria [3–5, 9, 11, 13, 19–21, 26–32, 35].

**Fig. 2:**
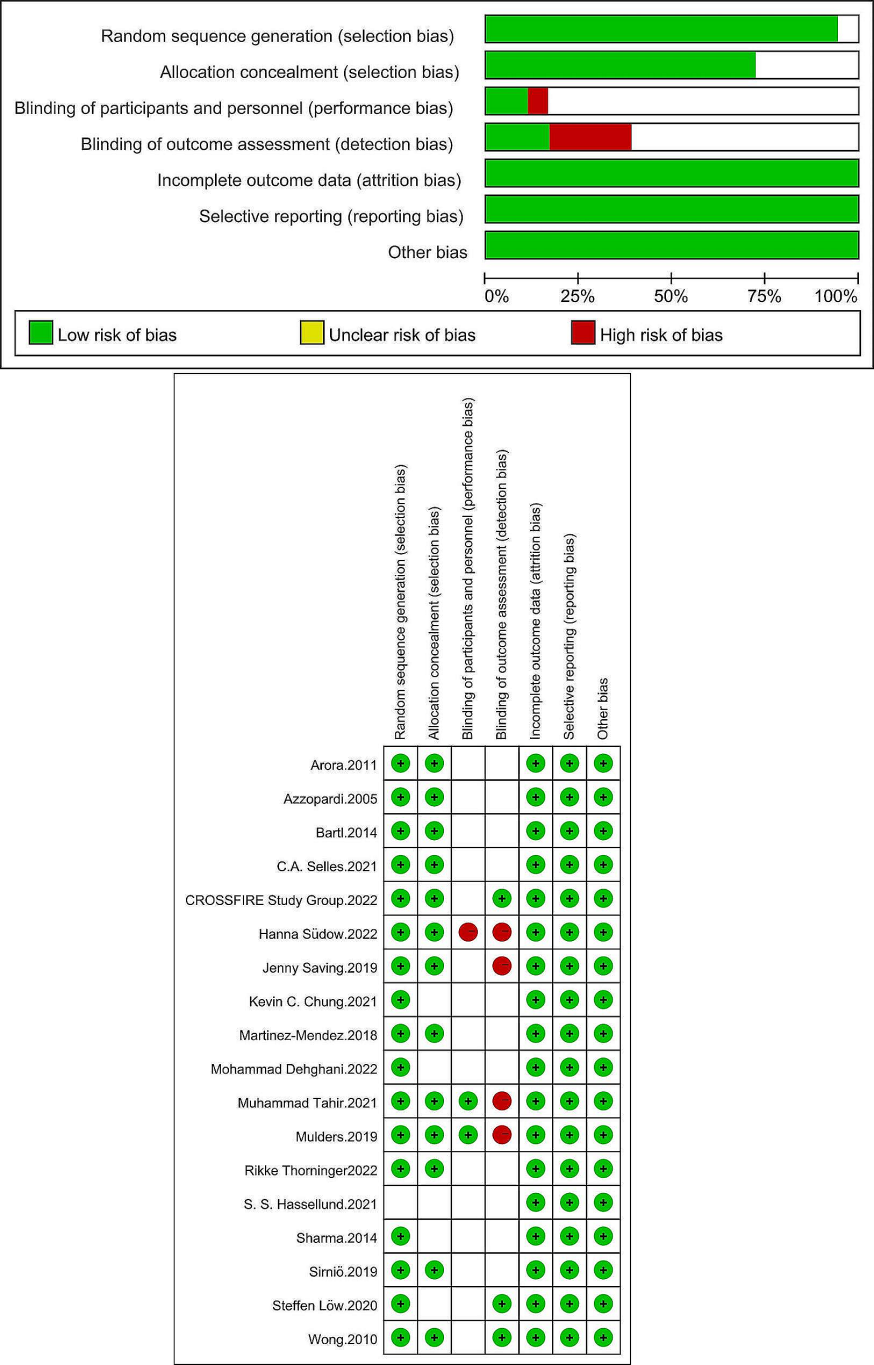
Characteristics and methodological quality of the included studies

The demographic characteristics of the patients in the seventeen studies are presented in Table 1. A total of 1730 patients were included due to loss of follow-up and other reasons: 862 in the nonsurgical group and 868 in the surgical group. Study characteristics were described in all seventeen studies in Fig. 2.

**Table 1:** Study characteristics

No.	Author and publication date	Study design	Sample size	Treatment		Mean age(years)		follow-up(m)
				*N*-S	S	*N*-S	S	
1	Azzopardi.2005	RCT	54	27	27	71 ± 9	72 ± 8	12
2	Martinez-Mendez.2018	RCT	97	47	50	70 ± 7	67 ± 8	29
3	Mulders.2019	RCT	92	44	48	60	59	12
4	Simiö.2019	RCT	80	42	38	64	62	24
5	Arora.2011	RCT	73	37	36	77.4	75.9	12
6	Wong.2010	RCT	60	30	30	71	70	N-S19; S20
7	Sharma.2014	RCT	64	32	32	48.10 ± 10.30	52.39 ± 9.05	24
8	Bartl.2014	RCT	149	81	68	74.4 ± 7.1	75.3 ± 6.7	-
9	Rikke Thorninger.2022	RCT	85	42	43	74	75	12
10	Hanna Südow.2022	RCT	66	33	33	76 ± 4.75	78 ± 5	36
11	CROSSFIRE Study Group	RCT	166	85	81	71.3 ± 7.6	70.5 ± 7.0	12
12	Jenny Saving.2019	RCT	119	63	56	78 ± 7	80 ± 5	12
13	C.A. Selles.2021	RCT	90	46	44	59 ± 10.37	62 ± 12.59	12
14	S. S. Hassellund.2021	RCT	100	50	50	73.9 ± 5.75	73.4 ± 6.5	12
15	Mohammad Dehghani.2022	RCT	50	25	25	33.72 ± 6.74	37.68 ± 9.18	24
16	Muhammad Tahir.2021	RCT	159	72	87	81 ± 2	81 ± 3	12
17	Kevin C. Chung.2021	RCT	94	44	50	74.3 ± 10.6	67.1 ± 6.3	24
	Kevin C. Chung.2021	RCT	91	44	47	74.3 ± 10.6	69.7 ± 8.5	24
	Kevin C. Chung.2021	RCT	85	44	41	74.3 ± 10.6	69.7 ± 6.5	24

*N-S: Non-surgical group S: Surgical group

Thirteen studies reported DASH scores and were included in the analysis. Significant heterogeneity was detected when the data from the thirteen studies were pooled (*Ι*^*2*^ = 69%). Compared with nonsurgical group, the results showed a significant reduction in DASH score with surgical treatment. (*WMD* 3.98, 95% *CI* 2.00 to 5.95, *P* < 0.001, Table 2).

**Table 2:** The result of meta-analysis

	Group	*N*	WMD(95%CI)	*P*
DASH(points)		13	3.98(2.00,5.95)	< 0.001
	≥ 65	8	3.79(0.91,6.68)	
	< 65	5	4.45(2.24,6.84)	
Grip strength (%)		7	-6.60(-11.61,-1.60)	0.01
	≥ 65	6	-4.39(-8.65,-0.14)	
	< 65	1	-16.88(-19.04,-14.72)	
Grip strength (kg)		7	-1.83(-3.77,0.10)	0.06
	≥ 65	3	-0.84(-3.73,2.04)	
	< 65	4	-2.55(-5.21,0.11)	
Radial deviation(°)		9	-2.85(-7.69,2.00)	0.25
	≥ 65	4	1.31(0.01,2.61)	
	< 65	5	-6.33(-13.96,1.30)	
Radial inclination(°)		10	-3.31(-4.47,-2.15)	< 0.001
	≥ 65	7	-3.95(-5.44,-2.46)	
	< 65	3	-2.12(-3.39,-0.84)	
Radial length(mm)		3	-2.67(-3.58,-1.77)	< 0.001
Ulnar deviation(°)		9	-2.31(-5.72,1.11)	0.19
	≥ 65	4	-1.58(-3.89,0.74)	
	< 65	5	-2.87(-8.91,3.17)	
Ulnar variance(mm)		7	0.35(-1.08,1.79)	0.63
	≥ 65	6	0.63(-0.94,2.20)	
	< 65	1	-1.30(-2.13,-0.47)	
Volar title(°)		2	-3.21(-3.61,-2.82)	< 0.001
Range of wrist supination(°)		12	-3.10(-5.27,-0.92)	0.01
	≥ 65	7	-2.64(-5.97,0.70)	
	< 65	5	-3.86(-6.68,-1.03)	
Range of wrist pronation(°)		11	-1.62(-3.01,-0.22)	0.02
	≥ 65	7	-1.94(-4.52,0.64)	
	< 65	4	-1.80(-3.49,-0.11)	
Range of wrist extension(°)		9	0.04(-1.37,1.45)	0.96
	≥ 65	7	0.82(-0.53,2.17)	
	< 65	2	-2.55(-6.37,1.27)	
Range of wrist flexion(°)		11	-2.88(-6.26,0.49)	0.09
	≥ 65	7	-1.16(-5.64,3.32)	
	< 65	4	-6.48(-9.15,-3.80)	

*N: number WMD: weighted mean difference

Fourteen studies were included in grip strength analysis. The units of the research indicators were percentages for seven papers and kilograms for the other seven papers. Articles where grip strength is measured in kilograms, the test for heterogeneity demonstrated that significant heterogeneity existed among these studies (*Ι*^*2*^ = 60.83%), and a random-effects model was adopted. There was no significant difference between the nonsurgical and surgical groups. (*WMD* − 1.83, 95% *CI* -3.77 to 0.10, *P* = 0.06, Table 2). Articles where grip strength is measured in percentages, the test for heterogeneity demonstrated was also significant among these studies (*Ι*^*2*^ = 88.35%), and a random-effects model was adopted. The surgical group had a significantly greater than the non-surgical group. (*WMD* − 6.60, 95% *CI* -11.61 to -1.60, *P* = 0.01, Table 2).

Nine studies were included in radial deviation analysis. Significant heterogeneity was detected when the data from nine studies were pooled (*Ι*^*2*^ = 96.66%), and a random-effects model was adopted. There was no significant difference between the nonsurgical and surgical groups (*WMD* − 2.85, 95% *CI* -7.69 to 2.00, *P* = 0.25, Table 2).

Ten studies were included in radial inclination analysis. Significant heterogeneity was found when the data from ten studies were pooled (*Ι*^*2*^ = 86.12%,), and a random-effects model was adopted. The nonsurgical group had a significantly less radial inclination than the surgical group for all ten of these studies. (*WMD* − 3.31, 95% *CI* -4.47 to -2.15, *P* < 0.001, Table 2).

Three studies were included in radial length analysis. Significant heterogeneity was found when the data from the three studies were pooled (*Ι*^*2*^ = 65.51%), and a random-effects model was adopted. The results showed that radial length was significantly less in nonsurgical group than in surgical group (*WMD* − 2.67, 95% *CI* -3.58 to -1.77, *P* < 0.001, Table 2).

Nine studies were included in ulnar deviation analysis. Significant heterogeneity was detected when the data from the nine studies were pooled (*Ι*^*2*^ = 94.01%), and a random-effects model was adopted. There was no significant difference between the nonsurgical and surgical groups (*WMD* − 2.31, 95% *CI* -5.72 to 1.11, *P* = 0.19, Table 2).

Seven studies were included in ulnar variance analysis. Significant heterogeneity was found (*Ι*^*2*^ = 96.12%), and a random-effects model was adopted. There was no significant difference between the nonsurgical and surgical groups (*WMD* 0.35, 95% *CI* -1.08 to 1.79, *P* = 0.63, Table 2).

Two studies were included in volar title analysis. There was no significant heterogeneity when the data from the two studies were pooled (*Ι*^*2*^ = 0%). The nonsurgical group had a significantly less volar tilt than the surgical group. (*WMD* − 3.21, 95% *CI* -3.61 to -2.82, *P* < 0.001, Table 2)

Twelve studies were included in range of wrist supination analysis. Significant heterogeneity was found (*Ι*^*2*^ = 86.62%), and a random-effects model was adopted. The nonsurgical group had a significantly less range of wrist supination than the surgical group. (*WMD* − 3.10, 95% *CI* -5.27 to -0.92, *P* = 0.01, Table 2).

Twelve studies were included in range of wrist pronation analysis. Significant heterogeneity was found (*Ι*^*2*^ = 70.74%), and a random-effects model was adopted. The surgical group had a significantly greater range of wrist pronation than the non-surgical group. (*WMD* − 1.62, 95% *CI* -3.01 to -0.22, *P* = 0.02, Table 2).

Nine studies were included in range of wrist extension analysis. No significant heterogeneity was detected when the data from nine studies were pooled (*Ι*^*2*^ = 23.76%). There was no significant difference between the nonsurgical and surgical groups. (*WMD* 0.04, 95% *CI* -1.37 to 1.45, *P* = 0.96, Table 2).

Eleven studies were included in range of wrist flexion analysis. Significant heterogeneity was found (*Ι*^*2*^ = 83.33%), and a random-effects model was adopted. There was no significant difference between the nonsurgical and surgical groups. (*WMD* − 2.88, 95% *CI* -6.26 to 0.49, *P* = 0.09, Table 2).

In the age stratification section of this study, we found that for elderly patients aged 65 years and older, the surgical approach improved patients’ radial inclination and reduced patients’ DASH, radial deviation compared to the non-surgical approach. In patients younger than 65 years of age, the surgical approach not only increased radial inclination and ulnar variance, but also decreased the DASH score and improved the range of wrist supination, pronation, and flexion compared to the non-surgical approach. However, the ranges of wrist flexion, pronation, and supination, and radial deviation, inclination, length, and ulnar variance were within the normal range in both the surgical and non-surgical groups. Although there were statistical differences, none of these measures reached the minimal clinical important difference in terms of clinical function.

**Fig. 3:**
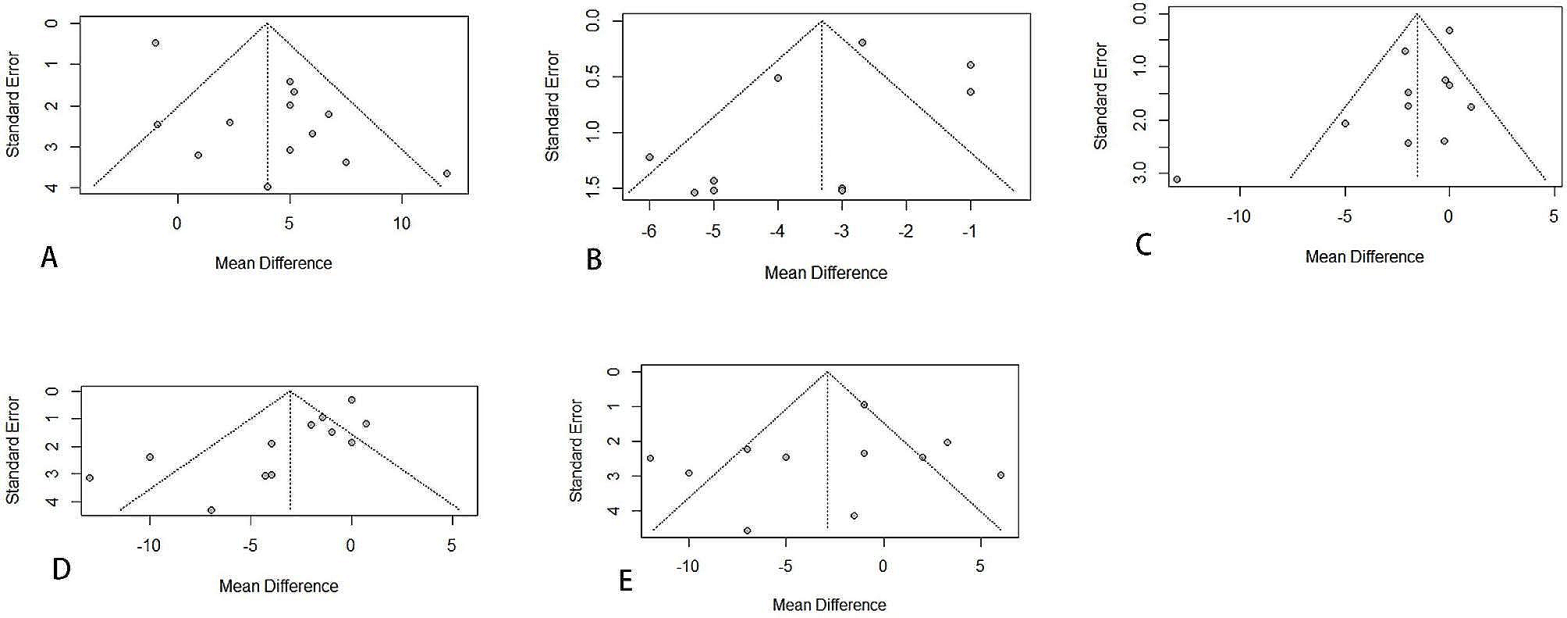
Funnel plot (**A**: DASH **B**: Radial inclination **C**: Range of wrist pronation **D**: Range of wrist supination **E**: Range of wrist flexion)

Funnel plot analysis of some studies was unable to be performed because of insufficient studies identified, as both visual examination and statistical analysis of funnel plots have limited power to detect bias if the number of trials is small. Therefore, we only performed funnel chart analysis for indicators with more than ten articles included in the literature (Fig. 3). The vertical line in the funnel plot represents the combined effect size (OR). Ideally, all studies should be evenly distributed on both sides of the vertical line in an inverted funnel shape, with the two diagonals representing the 95% confidence interval line. Due to the subjectivity of the funnel plot results, we conducted the egger test (Table 3), and we found that there was no publication bias in other studies except for the DASH score and supination study. In order to verify the stability of the DASH score and the supination result, we carried out sensitivity analysis of all studies with the leave-one-out method, and the study conclusion remained unchanged, which proved that our study was robust.

**Table 3:** Egger’s test

	t	*P*
DASH score	4.43	0.001
Radial inclination	-1.12	0.296
Range of wrist pronation	-2.10	0.065
Range of wrist supination Range of wrist flexion	-3.68 -0.77	0.004 0.461

## Discussion

This meta-analysis was performed to compare the functional and radiographic outcomes of the non-surgical and surgical treatment in distal radius fractures. There were significant differences in DASH score, as well as radial inclination, radial length, volar title, range of wrist pronation, range of wrist supination, range of wrist flexion, indicating better wrist function in surgical treatment group.

The current literature on the treatment of distal radial fractures is more controversial. Over the last 2 decades, there has been a movement toward open reduction and volar plate fixation [2]. Beharrie et al. reported better DASH scores and no loss of fracture reduction, and they recommended ORIF for elderly patients with distal radial fractures [6]. Kamano et al. evaluated on 33 patients who had displaced fractures of the distal radius that were treated with volar plating [17]. Nighty-7% excellent to good results according to the Gartland and Werley 12 rating scale. Jupiter et al. reported 90% excellent to good results with use of the PRWE score and the Physical Activity Scale in 20 patients who were managed with ORIF with use of palmar locking plates for the treatment of distal radius fracture [16]. Young et al. evaluated twenty-five patients for function and radiographic results following non-surgical treatment of displaced distal radius fractures [36]. The functional assessment revealed that 22 patients (88%) had excellent or good results and 3 (12%) had fair or poor results, indicating non-surgical treatment yields satisfactory outcome.

Comparative studies have been published to evaluate the outcome of surgical and non-surgical treatment in patients with distal radial fractures, but failed to reach a consensus. Martinez-Mendez et al. compared outcomes in elderly patients with intra-articular distal radius fractures treated by surgical and conservative treatment [21]. Over a 2-year follow-up, the functional outcomes and quality of life were significantly better after volar plating fixation compared with conservative treatment. They concluded that surgical plating leads to better outcomes than conservative treatment for patients with distal radius fractures. Bartl et al. compared ORIF with plaster immobilization in 149 patients, and found similar functional outcomes at 12 months [5]. They concluded that primary nonsurgical management is also effective in suitable patients. Arora et al. reported that the range of motion, the level of pain, and the PRWE and DASH scores were not different between the surgical and non-surgical treatment groups at the twelve-month follow-up examination [3].

Several meta-analyses have been published on the comparison between surgical and non-surgical treatment. Cui et al. compared internal versus external fixation for unstable DRFs, and the results showed that the open internal fixation was superior than external fixation regarding postoperative complications, clinical results and radiological outcomes [10]. Wei et al. also evaluated the external fixation and internal fixation for DRFs, and found the ORIF resulted in better follow-up outcome measures [34]. The result does not support the theory that surgical treatment can provide better clinical outcomes for DRFs. Ju et al. analyzed 8 studies with a total of 440 patients in the surgical groups and 449 in the control groups [15]. They concluded that surgical and nonsurgical managements produce similar results in the treatment of DRFS.

In the present meta-analysis, thirteen RCT trails were included in the analysis with 1278 patients to compare the DASH scores. We found a significant reduction in DASH score with surgical treatment groups. In addition to this, there was no significant improvement of grip strength in favor of surgical treatment. For the radiological outcomes, the surgical group had significantly greater radial inclination, radial length, and volar tilt than the surgical group. However, no difference was found in radial deviation, ulnar deviation, ulnar variance. For the wrist range of motion evaluation, there was no significant difference in range of wrist extension and range of wrist flexion between non-surgical and surgical treatment groups, which is in accordance with those of Chen et al [8]. But there was a little difference in range of wrist supination between those two different treatments.

In the age stratification section of this study, we found that for elderly patients aged 65 years and older, the surgical approach improved patients’ radial inclination and reduced patients’ DASH, radial deviation compared to the non-surgical approach. In patients younger than 65 years of age, the surgical approach not only increased radial inclination and ulnar variance, but also decreased the DASH score and improved the range of wrist supination, pronation, and flexion compared to the non-surgical approach. Therefore, we suggest that the surgical approach should be considered first for patients with distal radius fractures.

The advantage of this meta-analysis is that all the included studies were prospective RCTs. However, potential limitations in this study need to be considered: (1) The number of studies meeting the inclusion criteria was small, and the follow-up time of the studies also varied; (2) No analysis of different types of distal radius fractures or different types of surgical intervention was performed; (3) Complications associated with surgery (e.g. infection, symptomatic hardware, peri-implant fracture, non-union, malunion, extensor and flexor tendon injuries) and those associated with non-operative management (nonunion, conversion to surgery, attritional tendon injuries) may have not been reported in the results of included studies. (4) Some unpublished studies and data can’t be included in this analysis. These limitations should be avoided in the design of future trials.

The results of this meta-analysis suggest that surgical treatment of distal radius fractures improved the DASH score and grip strength compared with nonsurgical treatment. Based on the present meta-analysis, we suggest that surgical treatment might be more effective in some patients with distal radius fracture. More RCT trails and individual patient data meta-analyses are needed for more precise conclusions.

## Conclusion

The results of this meta-analysis suggest that some patients with surgical treatment of distal radius fractures not only decreased the DASH score, but also improved the range of wrist pronation and the range of wrist supination compared with nonsurgical treatment. Based on the present meta-analysis, we suggest that surgical treatment might be more effective in some patients with distal radius fracture.

### Electronic supplementary material

Below is the link to the electronic supplementary material.

Supplementary Material 1

## Data Availability

All data generated or analysed during this study are included in this published article.

## Declarations

### Registration and protocol

The review is not registered currently and we will complete it as soon as possible.
